# Implementing Non-Pharmacologic Interventions for People Living with Dementia in Long-Term Care Settings

**DOI:** 10.1093/geroni/igaa057.2420

**Published:** 2020-12-16

**Authors:** Emily Ihara

## Abstract

Person-centered dementia care is a best practice recommendation by the Alzheimer’s Association, and non-pharmacologic interventions that emphasize well-being and quality of life as defined by the individual are important to preserve personhood. Non-pharmacologic, person-centered interventions have been shown to effectively address various neuropsychiatric symptoms, commonly known as behavioral and psychological symptoms in dementia (BPSD), which include a wide range of behaviors such as verbal or physical aggression, agitation, wandering, and pacing. Interventions that are focused on an individual’s holistic needs and preferences can stimulate positive emotions and behavior regardless of the stage of dementia. Person-centered care emphasizes a social model of care, rather than a medical model, by focusing on an individual’s emotional needs and care preferences that are consistent with their previous lifestyle. This symposium explores four different non-pharmacologic interventions for individuals living with dementia and discusses challenges and best practices for implementation in long-term care settings. For example, a best practice includes “buy-in” from facility staff who ultimately are responsible for implementing interventions that follow a social care model. A challenge found includes creating consistency and adherence to non-pharmacologic interventions so they are sustained over time, potentially replacing additional doses of medication. Symposium presenters will discuss the Mason Music & Memory Initiative (M3I), the Alzheimer’s Poetry Project, Birdsong, and TimeSlips, which are all interventions that are relatively low-cost and easy to implement by non-specialists. Strategies for intergenerational programming and adaptability of these programs to different contexts will also be discussed.

